# Supplementary material for: Integrative Analysis of Metabolomic and Transcriptomic Data Reveals the Mechanism of Color Formation in Corms of Pinellia ternata
Source: Int J Mol Sci. 2023 Apr 28;24(9):7990. doi: 10.3390/ijms24097990 (PMC10178707; doi:10.3390/ijms24097990)
Supplement: Supplementary file 1 [file ijms-24-07990-s001.zip › Figure S1.pdf]

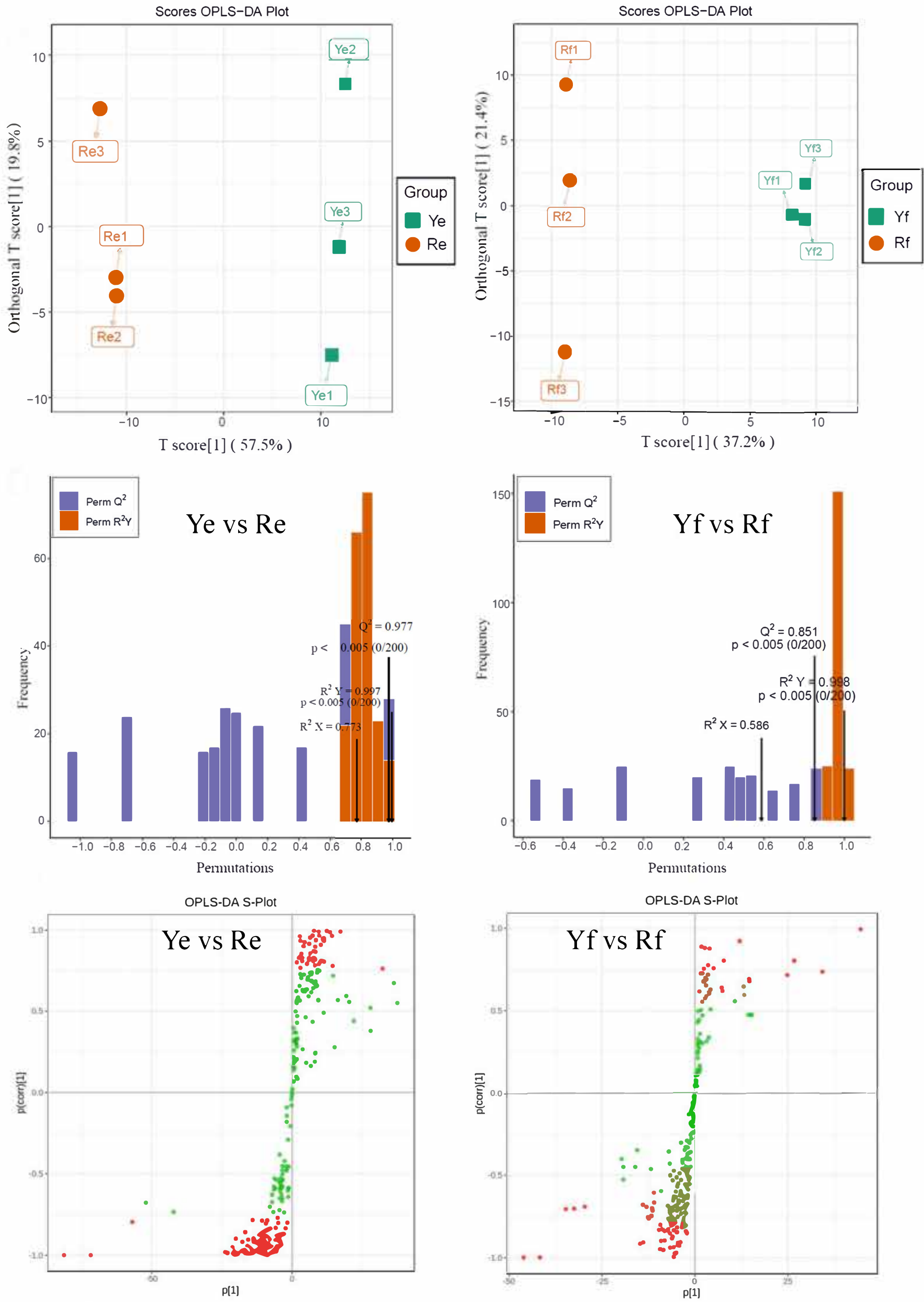

Figure S1. Orthogonal partial least squares-discriminant analysis (OPLS-DA) in epidermis and flesh of red and yellow corms.
