# Supplementary figures and images for: Integrative Analysis of Metabolomic and Transcriptomic Data Reveals the Mechanism of Color Formation in Corms of Pinellia ternata
Source: Int J Mol Sci. 2023 Apr 28;24(9):7990. doi: 10.3390/ijms24097990 (PMC10178707; doi:10.3390/ijms24097990)

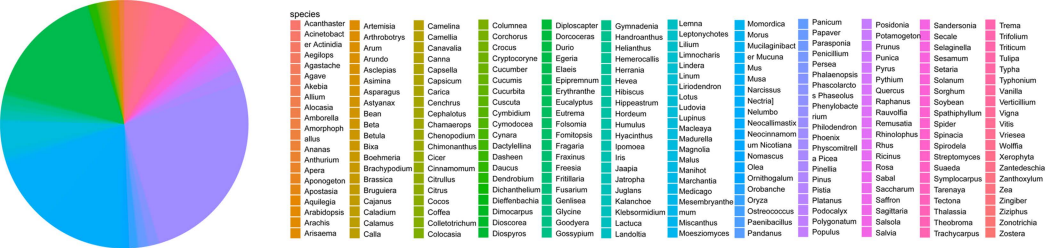

Figure S2. All transcripts were assigned to database Nr.

Supplement: Supplementary file 1 [file ijms-24-07990-s001.zip › Figure S2.pdf]

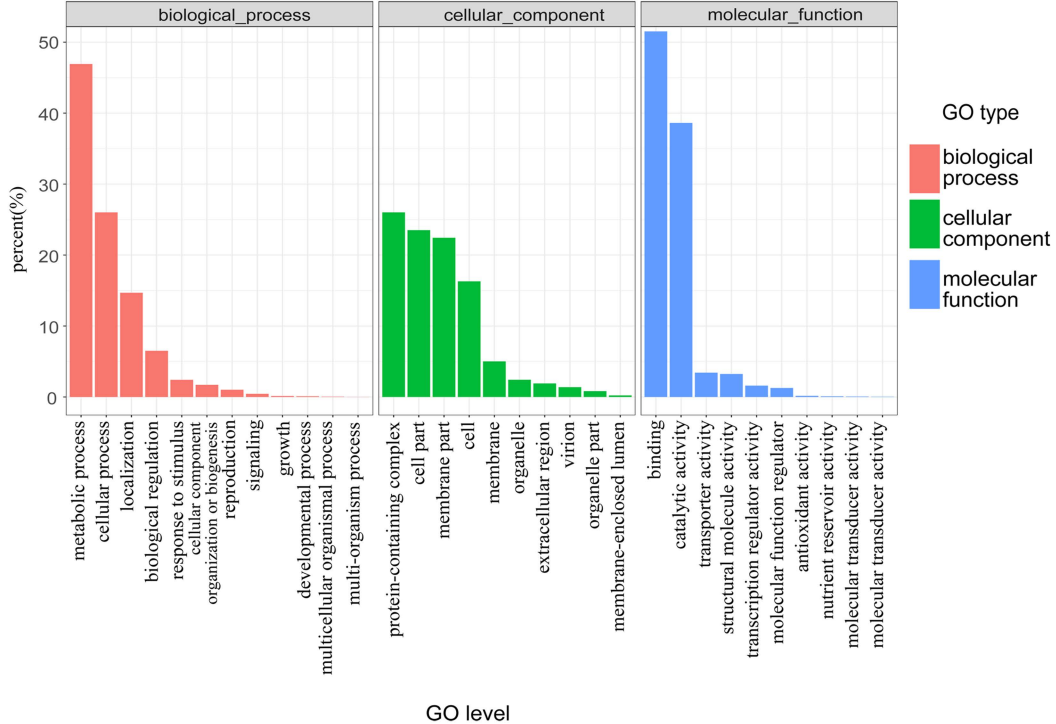

Figure S4. All transcripts were assigned to database GO.

Supplement: Supplementary file 1 [file ijms-24-07990-s001.zip › Figure S4.pdf]

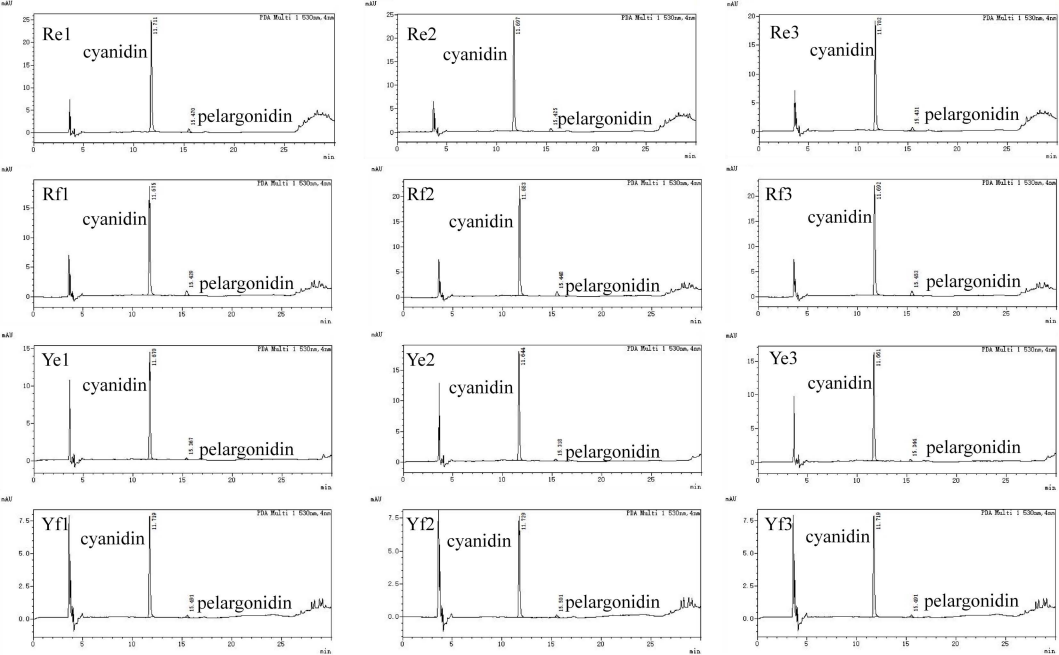

Figure S6. HPLC profile of 12 samples.

Supplement: Supplementary file 1 [file ijms-24-07990-s001.zip › Figure S6.pdf]
